# COVID‐19 and programmatic assessment

**DOI:** 10.1111/tct.13207

**Published:** 2020-06-26

**Authors:** Laksha Bala, Cees van der Vleuten, Adrian Freeman, Dario Torre, Sylvia Heeneman, Amir H Sam

**Affiliations:** ^1^ Faculty of Medicine Imperial College London London UK; ^2^ Department of Educational Development and Research Faculty of Health, Medicine and Life Sciences School of Health Professions Education Maastricht University Maastricht the Netherlands; ^3^ University of Exeter Medical School Exeter UK; ^4^ Department of Medicine F. Edward Hébert School of Medicine Uniformed Services University Bethesda Maryland USA; ^5^ Department of Pathology Faculty of Health, Medicine and Life Sciences School of Health Professions Education Maastricht University Maastricht the Netherlands

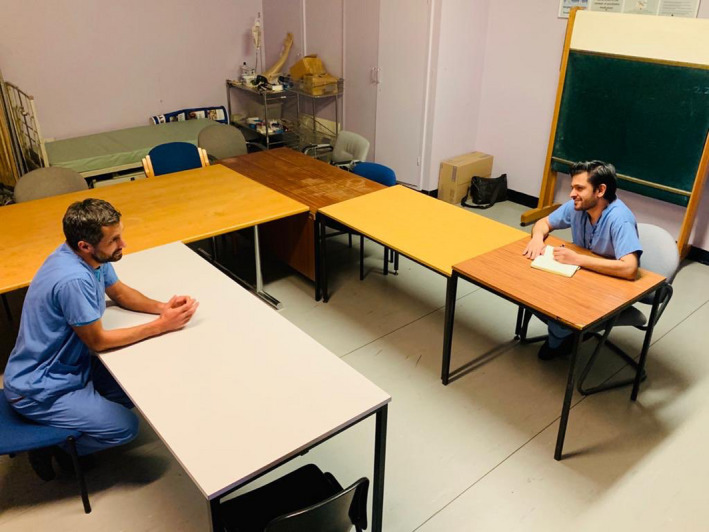

As a result of the COVID‐19 pandemic, medical schools across the world have been faced with the unprecedented situation of closing their doors and cancelling clinical teaching and examinations. The traditional model of assessment relying on high‐stakes clinical examination(s) is no longer possible, nor fully fit for purpose. How can medical schools still demonstrate that their students have reached the threshold for safe practice and are able to graduate? A potential solution for this issue is programmatic assessment.

Programmatic assessment involves the longitudinal collection of information about a student based on multiple low‐stakes assessments.^1^ The purpose of each individual assessment is to provide feedback, not to pass or fail a learner. By assembling feedback from many assessments, the learner monitors their progress and discusses this with a mentor. High‐stakes decisions are based on all of the data and are performed by an independent competence committee. Programmatic assessment moves away from an end‐of‐course ‘big bang’ examination to a continuous approach of assessment that drives learning in a meaningful and self‐directed way.

Programmatic assessment moves away from an end‐of‐course ‘big bang’ examination to a continuous approach of assessment that drives learning …

During the COVID‐19 pandemic, medical schools have used a variety of workplace‐based assessments of clinical and professional skills, as well as applied knowledge tests to collect information on learning and performance. We propose that programmatic assessment could be used in a hybrid model to complement more conventional methods of assessment to make high‐stakes decisions.

… programmatic assessment could be used in a hybrid model to complement more conventional methods of assessment …

Programmatic assessment has been gaining traction for many years, but its implementation is often hindered by challenges around nationwide ranking, university regulations, reliability and a resistance to change from the traditional model of high‐stakes, summative examinations. With the cancellation of clinical examinations and restricted student access to clinical sites, medical schools have been forced to consider alternative ways to evidence their students’ progression. There is a strong evidence base for the benefits of programmatic assessment, including studies demonstrating the ability to facilitate learning and maximise the robustness of high‐stakes decisions,^2^ as well as identify students at risk of poor academic progress, and thereby optimise timely interventions.^3^

The sole use of high‐stakes, summative examinations has been described as intrinsically flawed because the ‘ideal’ assessment, capable of assessing all of the necessary competencies, does not exist, and placing an emphasis on single high‐stakes examinations may promote poor learning styles.^2^

Although concerns have been raised regarding the reliability of ‘subjective’ single assessment points, it has been shown that acceptable reliability is achievable through large numbers of assessment points, varying methods of assessment (including both standardised and non‐standardised assessment) and multiple assessors.^4^

Although this is a challenging time for medical education, the COVID‐19 crisis may in fact present an opportunity for reflection and adaptation. Here are a few considerations regarding programmatic assessment during the COVID‐19 pandemic.

- A number of low‐stakes assessments (e.g. case‐based exercises) can still be carried out remotely and optimised for feedback.
- The collection of several workplace‐based assessments may not be possible, e.g. the direct observation of a student encounter with a real patient in a hospital setting. We could, however, use results of direct observations over the past 6 months across several rotations, triangulate those with other collected data points for the same student, and use these to come to a progress decision regarding the student's learning.
- In the situation that a final summative exam is required, for example for graduation, the longitudinal features of programmatic assessment could still be used in a hybrid model to reach a high‐stakes decision. Studies have shown that in programmatic assessment, the data gathered over time after proper aggregation can be trusted to make a decision.^5^
- High‐stakes decisions can be made by a committee of assessors using secure online communication software. Previously collected and electronically stored assessments can be accessed remotely, compiled, and prepared for a committee to be examined and discussed in a secure online environment.
- The use of programmatic assessment in these challenging times can be compared with the follow‐up assessment of a patient in a clinic. The opportunity to examine and reassess a patient and their progress on multiple occasions, compared with a single assessment, would offer more evidence to support any decisions made for that patient.

Having assessment data over time may help to ease some of our angst about learners’ assessment decisions during such unprecedented times. With the dissemination of this message, medical schools may overcome their apprehension regarding programmatic assessment and recognise its many benefits.

In the face of adversity, we have stumbled upon a unique opportunity to enrich students’ learning, and in the words of Winston Churchill, we should ‘never let a good crisis go to waste’.
